# A 3D-Printed Educational Model for First-Line Management of BPPV in Emergency Departments

**DOI:** 10.3390/audiolres14060086

**Published:** 2024-12-02

**Authors:** Pietro Canzi, Elena Carlotto, Stefania Marconi, Silvia Quaglieri, Giuseppe Attanasio, Francesca Yoshie Russo, Ilaria Ottoboni, Silvia Ponzo, Andrea Scribante, Stefano Perlini, Marco Benazzo

**Affiliations:** 1 Department of Clinical, Surgical, Diagnostic and Pediatric Sciences, University of Pavia, 27100 Pavia, Italy; 2 Department of Otorhinolaryngology, Fondazione IRCCS Policlinico San Matteo, University of Pavia, 27100 Pavia, Italy; 3 Department of Civil Engineering and Architecture, University of Pavia, 27100 Pavia, Italy; 4 Department of Sense Organs, Sapienza University of Rome, 00100 Rome, Italy; 5 Department of Otolaryngology, S. Croce Hospital, 12100 Cuneo, Italy; 6 Unit of Orthodontics and Pediatric Dentistry, Section of Dentistry, Department of Clinical, Surgical, Diagnostic and Pediatric Sciences, University of Pavia, 27100 Pavia, Italy; 7 Unit of Dental Hygiene, Section of Dentistry, Department of Clinical, Surgical, Diagnostic and Pediatric Sciences, University of Pavia, 27100 Pavia, Italy; 8 Emergency Medicine, Vascular and Metabolic Disease Unit, Department of Internal Medicine, Fondazione IRCCS Policlinico San Matteo, University of Pavia, 27100 Pavia, Italy

**Keywords:** benign paroxysmal positional vertigo, 3D printing, emergency department, training, labyrinth, tangible model, maneuver, posterior semicircular canal

## Abstract

Background: We present a feasibility study on the development of a 3D-printed (3DP) model of benign paroxysmal positional vertigo (BPPV) and its validation as an educational tool for training in therapeutic maneuvers. Methods: A 1.5:1 3DP model of the human labyrinth, supplemented by a 1:1 3DP model of the skull, was obtained from a computed tomography scan. We presented the model to 15 Emergency Medicine residents, 15 medical students, 15 Otolaryngology residents, and 15 Otolaryngology practitioners from two academic referral centers. Participants performed the Semont and Epley maneuvers on the model twice, once before and once after observing the biomechanics of BPPV using this tool. A specific survey was then administered to assess both performance improvement and satisfaction. Results: All the trainees demonstrated an improving trend on the second attempt. The medical students ameliorated significantly after the training in both Epley (*p* = 0.007) and Semont maneuvers (*p* = 0.0134). The Emergency Medicine residents improved significantly in Semont maneuvers (*p* = 0.0134). Self-reported understanding of the BPPV mechanics improved significantly after training in all the groups (*p* < 0.05). Conclusions: The preliminary data highlighted the potential benefits of training on the 3DP model for practitioners involved in the first-line management of BPPV.

## 1. Introduction

Benign paroxysmal positional vertigo (BPPV) is one of the most common causes of vertigo [1]. It accounts for a high proportion of both emergency department admissions and ENT outpatient visits. The management of this condition can be a concern for clinicians, particularly in the emergency setting, as the differential diagnosis includes potentially life-threatening causes of vertigo [2,3]. Understanding the underlying biomechanisms of the BPPV is essential for its correct identification and first-line treatment [4]. The recent literature has suggested that the didactic method for BPPV could be innovated [5,6,7,8,9] and has shown a growing interest in the development of explanatory models of BPPV physiopathology [10,11,12]. Thanks to technological advances, studies on dedicated mobile applications [13,14] and digital or multimedia approaches are flourishing [15,16,17]. In addition, papers proposing educational tools for the management of BPPV are emerging on the international scene [18,19]. Meanwhile, three-dimensional (3D) printing technology has gained a central role in the production of anatomical models with an increasing impact on healthcare, including medical education [20]. Today, the usefulness of 3D-printed (3DP) models in BPPV training is an emerging topic, and its potential benefits need to be explored, starting with the most common BPPV variants. The aim of this study is to create a 3D-printed (3DP) tangible model of the inner ear for educational purposes. The model is intended to facilitate understanding of the mechanisms of BPPV therapeutic maneuvers, particularly for the most common form involving the posterior semicircular canal (PSC). Firstly, we investigated the feasibility of a tangible model produced entirely using 3D printing technology. Second, we aimed to validate the model as an educational tool for training in BPPV therapeutic maneuvers. It is aimed at emergency medicine (EM) residents, otolaryngology (ENT) residents, and medical students. To achieve this purpose, we quantified the trainees’ improvement in the PSC BPPV management after the educational session and the satisfaction of the participants. Preliminary impressions and further applications of this simulation tool are discussed, with particular reference to first-line BPPV management in the emergency setting.

## 2. Materials and Methods

### 2.1. Model Creation

**Figure 1:**
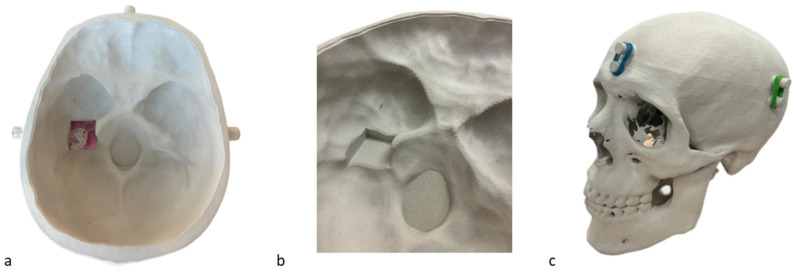
Skull 3DP model. (**a**) Lower part of the model; (**b**) zoom on the artificially created inlet, in the left side of the skull; (**c**) closure of the two parts of the skull through rubber bands.

The human skull 3DP model. A 1:1 3D model of a human skull was obtained from a high-resolution Computed Tomography (HRCT) scan of an anonymized patient with normal anatomy who had given his informed consent. The HRCT data underwent an image segmentation process to extract the structure of interest and save it as a discretized representation (Standard Tessellation Language file format) suitable for 3D printing. The model was then printed using the HP MultiJet Fusion 580 Color 3D Printer (HP Inc., Palo Alto, CA 94304 USA). The material employed was nylon PA12 powder. The model was realized in two parts: a top and a bottom part, in order to visualize the internal structures present in the bottom part of the model. An inlet was artificially created in the left side of the lower cranial cavity to accommodate the labyrinth model (Figure 1a,b). The two parts of the skull were closed during the simulation of therapeutic maneuvers. The closure of the two parts was achieved by printing 4 artificial spikes on each side. Four elastic bands were used to connect the 2 parts of the skull through their respective spikes (Figure 1c).

**Figure 2:**
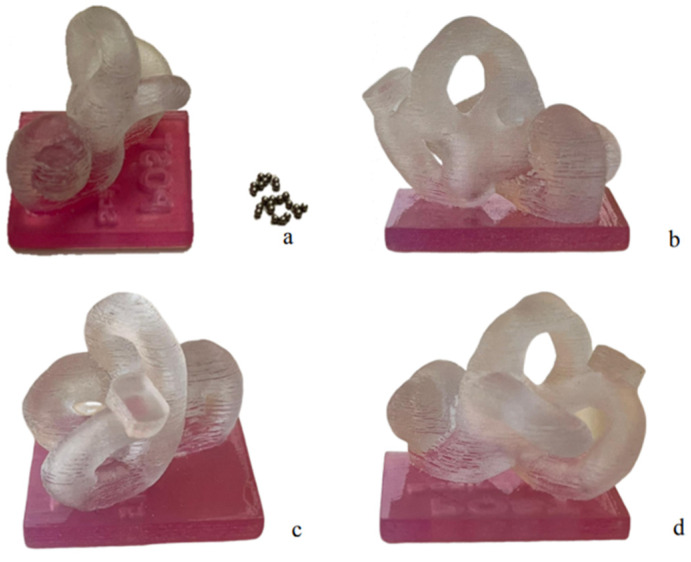
Labyrinth 3DP model. The 3DP model of a left labyrinth, seen anteriorly with 1 mm steel balls (**a**), medially (**b**), posteriorly (**c**), and laterally (**d**).

The labyrinth 3DP model. The J750 Digital Anatomy (Stratasys, Eden Prairie, MN 55344, USA) 3D printer, implementing material jetting technology, was used to produce the 1.5:1 left labyrinth model. The material used to create the labyrinth structure itself was Stratasys’ VeroClear (RGD810), a transparent rigid photopolymer resin. A support structure was designed to ensure the correct anatomical orientation of the labyrinth and to allow the model to be inserted into its inlet in the skull. A mixture of VeroClear and Stratasys’ VeroMagenta (RGD851)—a photopolymer resin with the same mechanical properties as VeroClear but a different color—was used to create the base (Figure 2). Stainless steel balls 1 mm in diameter were used to simulate the otoliths (Figure 2a). The canals were smoothed to improve the transparency of the model and provide a more satisfactory view. Sandpaper and a commercially available transparent polishing spray were used for smoothing. The labyrinth model was printed with a hollow PSC and vestibule, while all other structures were solid. Two holes—one in the upper part of the PSC at the non-ampullary arm and the other in the vestibule—were made. The former was intended to be the entrance for the steel balls into the model, while the latter was intended to let the steel balls out of the labyrinth, thus representing their correct arrival in the vestibule. To this end, a plug was printed in the same material as the labyrinth model to temporarily close the hole in the canal. It was shaped to prevent extrusion of the steel balls and protrusion into the lumen of the canal.

### 2.2. The Training-Experience

A total of 60 participants from two tertiary academic centers were enrolled: 15 EM residents, 15 medical students, 15 ENT residents, and a control group of 15 ENT practitioners. Each participant was given a graphic description of both the Semont and Epley maneuvers, explaining all the steps required to perform them correctly. The schematic images provided by Bhattacharyya and colleagues [1] were used for this purpose. The participants performed both maneuvers on the 3D-printed skull, with the upper part of the skull closing the model and thus concealing the inner ear. Two steel balls representing loose otoconial debris were previously inserted into the labyrinth model. After each maneuver, the upper part of the skull model was removed, and the position of the steel balls was checked by our team. The presence of the steel ball still within the PSC channel was considered a failure, whereas the presence of the steel ball either within the vestibule or outside the labyrinth model was considered a success. The labyrinth model was shown in detail to the participants. The underlying mechanisms of the Epley and Semont maneuvers were explained, particularly in relation to cranial movements. For example, the specific angles required for a successful outcome were demonstrated in detail using the tangible model. Participants were encouraged to ask questions about the anatomy, pathophysiology, and therapeutic mechanics of the maneuvers. They also had the opportunity to handle the model and observe the movement of the steel balls inside the PSC. For the second time, the participants performed the Epley and Semont maneuvers with the skull model covered. The same rules as before were used to assess the success or failure of the maneuvers. After training with the 3DP model, a questionnaire was administered anonymously to each participant (Table 1). The questionnaire consisted of four questions (1a, 1b, 2a, 2b) to quantitatively assess the effectiveness of the model for training purposes and five questions (3–7) to assess each student’s subjective perception of the usefulness of the experience. The last question (8) was about the group to which they belonged, which was the only identifying information.

### 2.3. Statistical Analysis

Statistical analyses were performed using the R software (R version 3.1.3, R Development Core Team, R Foundation for Statistical Computing, Wien, Austria). Descriptive statistics were calculated for all groups (the mean and standard deviation). Kruskal–Wallis test was applied to determine whether significant differences existed between the groups. Dunn’s multiple comparisons test was used as a post-hoc evaluation. In addition to this, paired *t*-test was used to compare the results of Questions 3 and 5. The limit for statistical significance for all statistical tests was predetermined at *p* < 0.05.

## 3. Results

### 3.1. Feasibility of 3DP BPPV Model

The 3DP BPPV model obtained from the HRCT scan was found to be feasible. The labyrinth model proved to be suitable for the steel balls rolling in it. Once placed on the dedicated inlet in the skull model, it allowed proper simulation of otolith movement and easy retrieval of the steel balls.

### 3.2. Validation as Educational Tool for BPPV Skills Training

#### 3.2.1. Performance in Repositioning Maneuvers

- Epley maneuver

**Figure 3:**
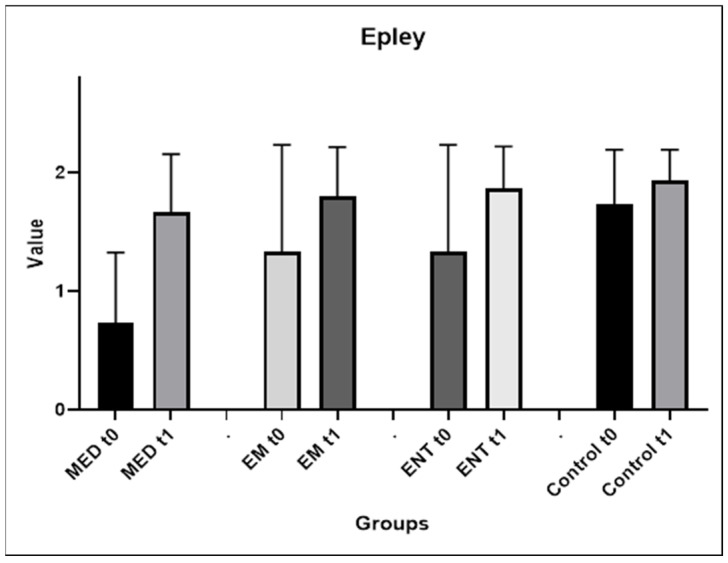
Evolution of the participants’ performances between the first and second attempts of the Epley maneuver. EM: EM residents, ENT: ENT residents, MED: medical students, t0: first attempt, t1: second attempt.

Figure 3 shows graphically the evolution of the participants’ performance between the first and second attempts. The medical students demonstrated a statistically significant improvement after the training, from a mean score of 0.73 (±0.59) to 1.67 (±0.49), *p* = 0.007. A trend toward improvement at the second attempt was also observed for the EM and ENT residents, although the results do not reach statistical significance. The mean scores increased from 1.33 (±0.90) to 1.8 (±0.41) and from 1.33 (±0.90) to 1.87 (±0.35) between the first and the second maneuver, for the EM and ENT residents respectively. The mean scores achieved by the control group were the highest compared to the other participants and varied very little between the first and second attempts, from 1.73 (±0.46) to 1.93 (±0.26). Comparisons between the groups showed a statistically significant difference between the results of the medical students and the control group on the first attempt (*p* = 0.0018). There was no statistically significant difference between the groups in the results obtained after training. Tables S1–S4 in the Supplementary Material show analytically the performance of the participants.

- Semont maneuver

**Figure 4:**
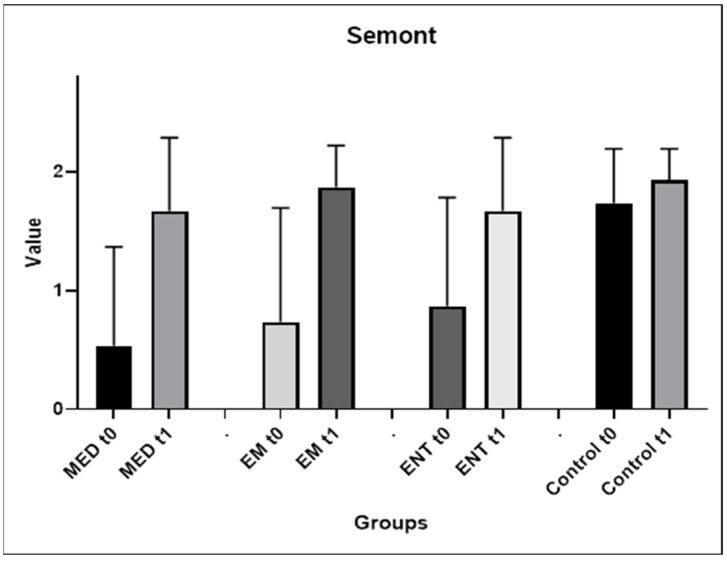
Evolution of the participants’ performances between the first and second attempts of the Semont maneuver. EM: EM residents, ENT: ENT residents, MED: medical students, t0: first attempt, t1: second attempt.

The performances on the Semont maneuver before and after training are shown in Figure 4. Mean scores increased significantly on the second attempt for both medical students, from 0.53 (±0.83) to 1.67 (±0.62), *p* = 0.0134, and EM residents, from 0.73 (±0.83) to 1.87 (±0.35), *p* = 0.0134. There was an improving trend for the ENT residents, from mean scores of 0.87 (±0.91) to 1.67 (±0.62), although this was not statistically significant. The highest scores were reported by the control group, 1.73 (±0.46) and 1.93 (±0.26) at the first and second attempts, respectively. Data on group scores are analytically described in Tables S1–S4 (Supplementary Material).

The comparisons between groups showed a statistically significant difference between the results of the medical students and the control group at the first attempt (*p* = 0.0078). There was no statistically significant difference between the groups in the results achieved after training.

#### 3.2.2. Satisfaction

- Question 3: How well would you rate your understanding of the mechanics behind the therapeutic maneuvers for BPPV before using the model?

**Figure 5:**
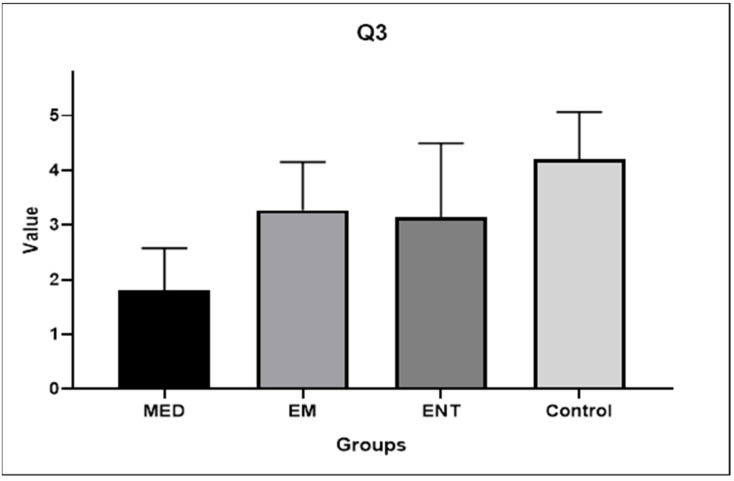
Participants’ answers to Question 3. EM: EM residents, ENT: ENT residents, MED: medical students.

The mean scores given by each group for Question 3 are illustrated in Figure 5. The lowest score was given by the medical students (mean score 1.80 ± 0.77) and the highest by the control group (mean score 4.2 ± 0.86). The response of the medical students was significantly different from that of all three other participant groups (*p* < 0.05).

- Question 4: How much do you think innovation in the educational approach to BPPV is needed to improve and facilitate understanding and effective implementation of diagnostic and therapeutic maneuvers?

**Figure 6:**
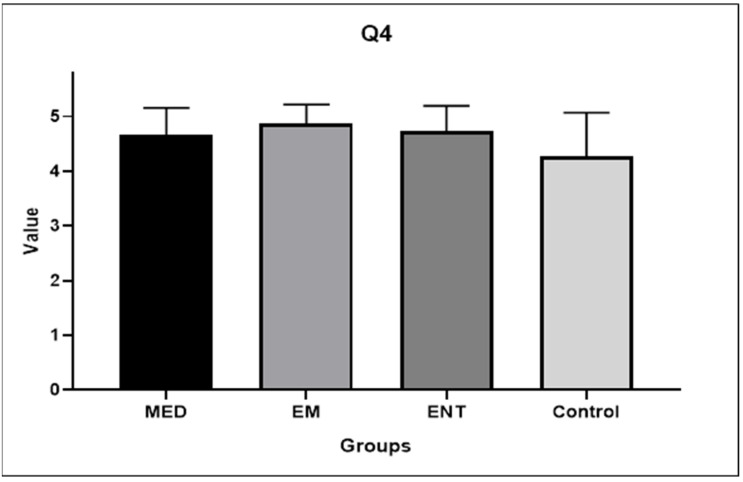
Participants’ answers to Question 4. EM: EM residents, ENT: ENT residents, MED: medical students.

All the participants attributed high scores to Question 4. The mean scores given by medical students, EM residents, ENT residents, and the control group were 4.67 (±0.49), 4.87 (±0.35), 4.73 (±0.46), and 4.27 (±0.80), respectively. Figure 6 shows graphically this data. There were no statistically significant differences among the groups.

- Question 5: How well would you rate your understanding of the mechanics behind the therapeutic maneuvers for BPPV after using the model?

**Figure 7:**
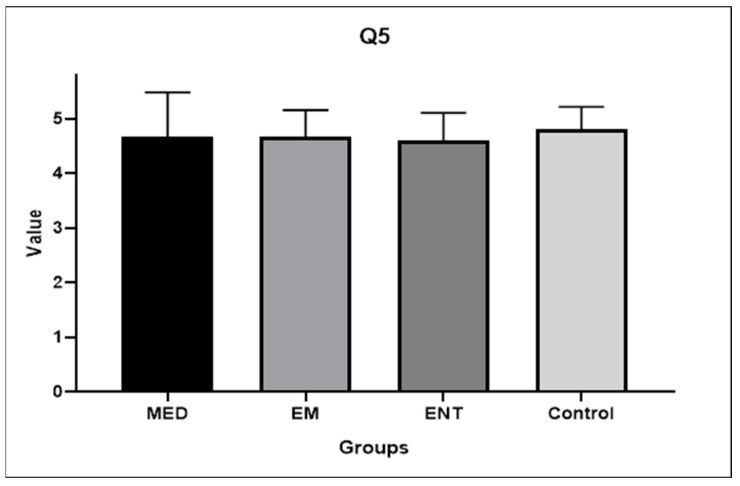
Participants’ answers to Question 5. EM: EM residents, ENT: ENT residents, MED: medical students.

There were no statistically significant differences in the responses between the groups: 4.67 (±0.82) for medical students, 4.67 (±0.49) for EM residents, 4.6 (±0.51) for ENT residents, and 4.8 (±0.41) for the control group, respectively. Figure 7 shows the above results.

The comparison between the answers to Question 3 (understanding of BPPV biomechanics before training) and 5 (understanding after training) showed a statistically significant difference in all cases. The mean scores increased from 1.8 ± 0.77 to 4.67 ± 0.81 (*p* < 0.001) for medical students, from 3.27 ± 0.88 to 4.67 ± 0.49 (*p* < 0.001) for EM residents, from 3.13 ± 1.36 to 4.6 ± 0.51 (*p* < 0.001) for ENT residents, and from 4.2 ± 0.86 to 4.8 ± 0.41 (*p* = 0.0025) for ENT practitioners, respectively.

- Question 6: How confident are you that you will be able to perform the maneuvers successfully on real patients after observing the mechanics in the 3DP model?

**Figure 8:**
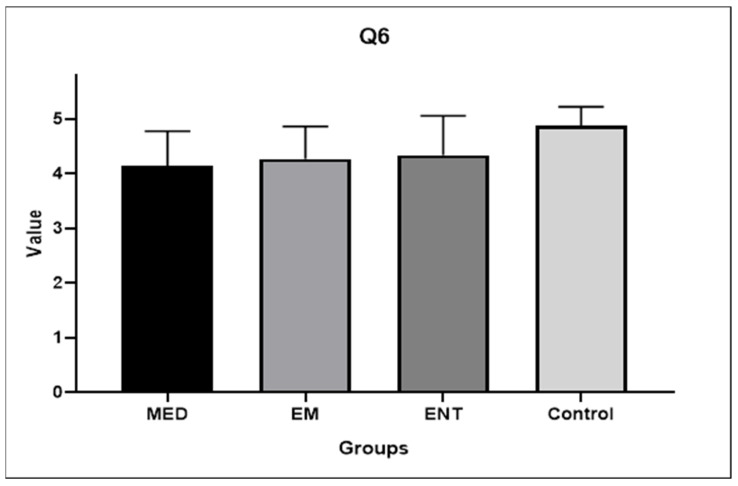
Participants’ answers to Question 6. EM: EM residents, ENT: ENT residents, MED: medical students.

Figure 8 shows the responses to Question 6 provided by each group. The lowest rating was given by medical students (mean score 4.13 ± 0.64), and the highest by the control group (mean score 4.87 ± 0.35). There is a statistically significant difference between the answers of the two groups mentioned above (*p* = 0.0071). In addition, the score of the control group was significantly higher than that of the EM residents (mean score 4.27 ± 0.59), *p* = 0.0365.

- Question 7: Which category of students do you think would benefit most from 3DP model-based learning?

**Figure 9:**
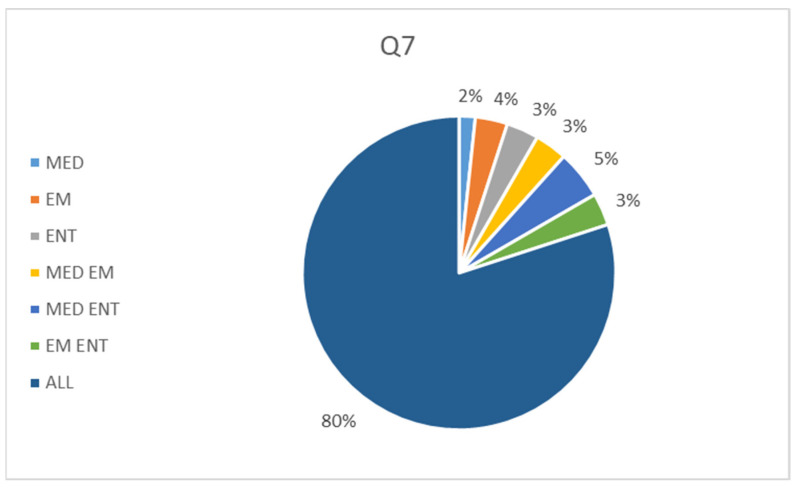
Participants’ answers to Question 7. EM: EM residents, ENT: ENT residents, MED: medical students.

The pie chart in Figure 9 shows the distribution of responses to Question 7. The 80% of participants believe that all categories of trainees would benefit from a 3DP BPPV model. The minority of respondents (2%) said that the tool could only be useful for medical students, with the remainder identifying one of the other groups or combinations as the beneficiary. 

The data of the satisfactory survey are presented in the Supplementary Material, Tables S5–S8.

## 4. Discussion

Although repositioning procedures are well established as the treatment of choice for BPPV, many authors have claimed that delays in performing therapeutic maneuvers are common [1]. Given the remarkable prevalence of BPPV, its proper management in emergency settings is desirable to reduce its health and social impact. Indeed, immediate treatment would avoid unnecessary diagnostic tests or inappropriate medications and facilitate the return of patients and caregivers to their daily activities. We therefore believe that the role of the EM practitioners should be strengthened in this regard, especially in centers where the otolaryngologist is not always present. Memorizing the steps of the maneuvers can be challenging, especially if the underlying mechanism is not understood. In fact, several authors have described 3D virtual simulation models intended for the visualization of otolith movements during maneuvers [11,21,22,23,24,25,26,27,28,29,30]. Despite the undoubted usefulness of these tools, we believe that handling a tangible model would allow trainees to practice with BPPV gestures. 

3D printing technology appears to be particularly suitable for this purpose, as it enables the rapid and cost-effective production of bespoke 3D objects of geometric complexity from easily manageable digital data [20]. The use of 3DP models is well established in otolaryngology, particularly for surgical training or individual surgical planning [31,32]. However, reports on the use of this technology for BPPV training are limited. There are only three experiences in the literature that bear any resemblance to ours. Fujisaka et al. developed a tangible head model with tenfold magnification of the two labyrinths. The authors report that their model is based on CT data but does not specify the technology used to obtain the final product. Their aim was to assess the effectiveness of the model in teaching the physical management of PSC BPPV. The study is based on a cohort of twenty medical students [33]. In addition, two recent publications have presented tangible models of labyrinths that were manufactured with a 3D-printed part and additional non-3DP elements. In both cases, the authors used the free, downloadable “Fluid-Filled Vestibular Apparatus for Vertigo Education” model design from Vestibular First, Philadelphia, PA (https://vestibularfirst.com/how-to-make-a-fluid-filled-vestibular-apparatus/ accessed on 8 August 2024) to create a 3DP core that was then assembled with a transparent silicone tubing system. Güneri and colleagues realized a partial 3DP model, which was greatly enlarged from its original size. The authors used it for BPPV conceptualization, mainly for the horizontal semicircular canals [34]. They presented videos in which the model was attached to the head of an actor who simulated the movements of the maneuvers. The authors claimed that this tool was useful for teaching, but they did not provide a practical training phase or a validation analysis. Meanwhile, Fontenot and colleagues used the same downloadable device as a visual aid to improve patients’ awareness of BPPV and ultimately reduce anxiety associated with symptoms [35]. 

The present work is actually a feasibility study for the development of the first fully 3DP model for BPPV skills training aimed at residents and students with a labyrinth close to the natural size. Our experience differs from the work of Güneri and colleagues by including a validation phase, measuring the improvement in maneuver performance, and collecting the preliminary impressions of the participants. Consistently with the experience of Fujisaka et al., our tool was designed to investigate the most common BPPV variant. However, our study included a larger cohort of not only medical students but also EM residents, ENT residents, and a control group of ENT practitioners. Furthermore, the authors had chosen a 10:1 ratio for their labyrinths, which we believe prioritized visibility over the accuracy of the repositioning gesture. In contrast, we have proposed a model that is closer to natural proportions and provides a more realistic experience for participants. The data derived from our work seems encouraging. Indeed, all the groups of trainees demonstrated an improving trend after the experience. The medical students ameliorated significantly in both Epley (*p* = 0.007) and Semont maneuvers (*p* = 0.0134), and the Emergency Medicine residents in Semont maneuvers (*p* = 0.0134). It could be observed that the medical students have benefited more from the training compared to the other groups. This can be explained by the fact that a higher number of EM, ENT residents, and ENT practitioners, had already achieved a score of 2/2 on the first maneuver attempt compared to medical students. As expected, the control group reported the highest results at the first attempt and the lowest improvement at the second one. After the 3DP model handling, the results achieved by the trainees were not statistically different among the groups and were comparable to those obtained by the ENT practitioners, demonstrating that adequate training can compensate for differences in theoretical background. Notably, despite the difference in initial level, all participants responded positively to the satisfaction survey (Figure 7). In fact, all the groups of trainees felt that their understanding of the biomechanics of BPPV had improved following their experience with the 3DP model. The control group also reported an improvement in comprehension. In this regard, it should be noted that not all ENT practitioners are equally prepared for the management of BPPV. It also confirms that BPPV is often managed mechanically without understanding its physiology. Instead, a proper comprehension of the underlying biomechanics is an added value when performing maneuvers. The starting point for this study was the perception that the didactic approach to the management of BPPV could be improved. The results of the survey seem to lead to the same conclusion. Actually, the participants unanimously agreed that an innovation in the educational approach to BPPV was necessary (Figure 6). Moreover, the great majority (80%) of the attendees considered the 3DP model-implemented learning useful for all categories of students and residents (Figure 9). The positive results recorded suggest that the 3DP model may be an appropriate training tool for developing the expertise required to manage BPPV in clinical practice. We believe that the implementation of the 3DP model educational session in EM clinic training would minimize return to specialist visits and delays in treatment, at least for the most common variants of BPPV.

However, there are a number of comments to be made about the present study. Firstly, it can be argued that the inertia of the movement of the steel balls within the model would be affected by the absence of a liquid medium to represent the endolymph. We recognize that this is a potential limitation, but we believe that our simplification meets the visualization objectives while avoiding some technical challenges. For example, the use of liquid medium would require the use of a moulded plug to temporarily close the hole in the vestibule. It would also be cumbersome to reposition the steel balls after each maneuver. Secondly, although the movement of the head during the maneuvers is not affected by the absence of a body, we can assume that this could lead to uncertainty or even inaccuracy in the performance of the maneuvers by the trainees. The introduction of a manikin attached to the skull should make the model more similar to everyday clinical practice, thus overcoming approximations in training. 

Overall, we believe that the positive results of this preliminary study should be corroborated with further experience aimed at optimizing the 3DP model and exploring its potential for other BPPV variants. 

## 5. Conclusions

This is the first study to demonstrate the feasibility of a tangible BPPV model created entirely using 3DP technology. In addition, it seems to be a valuable tool in the acquisition of specific skills for BPPV treatment. In our opinion, the evidence supported by this report should encourage the implementation of our 3DP model in the training of practitioners involved in the first-line management of BPPV, particularly in emergency departments. 

## Figures and Tables

**Table 1:** Questionnaire.

	Questions	Answers
1a	When performing the Epley maneuver, how many steel balls were you able to guide into the vestibule before directly observing their movement inside the 3DP model?	0 1 2
1b	When performing the Epley maneuver, how many steel balls were you able to guide into the vestibule after having directly observed their movement inside the 3DP model?	0 1 2
2a	When performing the Semont maneuver, how many steel balls were you able to guide into the vestibule before directly observing their movement inside the 3DP model?	0 1 2
2b	When performing the Semont maneuver, how many steel balls were you able to guide into the vestibule after having directly observed their movement inside the 3DP model?	0 1 2
3	How well would you rate your understanding of the mechanics behind the therapeutic maneuvers for BPPV before using the model?	1 2 3 4 5
4	How much do you think innovation in the educational approach to BPPV is needed to improve and facilitate understanding and effective implementation of diagnostic and therapeutic maneuvers?	1 2 3 4 5
5	How well would you rate your understanding of the mechanics behind the therapeutic maneuvers for BPPV after using the model?	1 2 3 4 5
6	How confident are you that you will be able to perform the maneuvers successfully on real patients after observing the mechanics in the 3DP model?	1 2 3 4 5
7	Which category of student do you think would benefit most from 3DP model-based learning? (more than one answer is possible)	EM ENT MED
8	To which of the following categories of trainees/control group do you belong?	EM ENT MED Control

For the questions from 3 to 6: 1 worst–5 better score. EM: EM residents, ENT: ENT residents, MED: medical students.

## Data Availability

Dataset available on request from the authors.

## Supplementary Materials

The following supporting information can be downloaded at: https://www.mdpi.com/article/10.3390/audiolres14060086/s1, Table S1: Results of medical students; Table S2: Results of EM residents; Table S3: Results of ENT residents; Table S4: Results of ENT practitioners; Table S5: Medical students’ answers to the satisfaction survey; Table S6: EM residents’ answers to the satisfaction survey; Table S7: ENT residents’ answers to the satisfaction survey; Table S8: ENT practitioners’ answers to the satisfaction survey.
