# Supplementary material for: A 3D-Printed Educational Model for First-Line Management of BPPV in Emergency Departments
Source: Audiol Res. 2024 Dec 2;14(6):1045–57. doi: 10.3390/audiolres14060086 (PMC11673195; doi:10.3390/audiolres14060086)
Supplement: Supplementary file 1 [file audiolres-14-00086-s001.zip › audiolres-3177404-supplementary.pdf]

Table S1. Results of medical students.

	Epley maneuvers		Semont maneuvers	
Participants	1st attempt	2nd attempt	1st attempt	2nd attempt
1	1	2	0	2
2	0	1	2	2
3	1	1	0	2
4	1	2	0	1
5	0	2	0	2
6	1	2	0	2
7	0	2	2	2
8	0	2	1	2
9	1	2	0	2
10	1	2	0	2
11	2	2	1	2
12	1	1	2	2
13	0	1	0	1
14	1	2	0	1
15	1	1	0	0

Results of the Epley and Semont maneuvers performed by the group of medical students before and after using the labyrinth model, expressed as the number of steel balls (from 0 to 2) brought to the labyrinth.

Table S2. Results of EM residents.

	Epley maneuvers		Semont maneuvers	
Participants	1st attempt	2nd attempt	1st attempt	2nd attempt
1	1	2	0	2
2	2	2	2	2
3	2	2	0	2
4	2	2	0	1
5	2	2	2	2
6	2	2	1	2
7	2	2	0	2
8	1	2	2	2
9	2	2	2	2
10	0	2	0	2
11	2	2	0	1
12	0	1	0	2
13	0	2	0	2
14	0	1	0	2
15	2	1	2	2

Results of the Epley and Semont maneuvers performed by the group of EM residents before and after using the labyrinth model, expressed as the number of steel balls (from 0 to 2) brought to the labyrinth.

Table S3. Results of ENT residents.

	Epley maneuvers		Semont maneuvers	
Participants	1st attempt	2nd attempt	1st attempt	2nd attempt
1	2	2	2	2
2	2	2	0	2
3	2	2	0	1
4	0	1	1	2
5	2	2	1	2
6	2	2	2	2
7	2	2	0	2
8	0	2	1	2

9	2	2	0	2
10	1	2	0	1
11	0	1	0	1
12	0	2	2	2
13	1	2	0	0
14	2	2	2	2
15	2	2	2	2

Results of the Epley and Semont maneuvers performed by the group of ENT residents before and after using the labyrinth model, expressed as the number of steel balls (from 0 to 2) brought to the labyrinth.

Table S4. Results of ENT practitioners.

	Epley maneuvers		Semont maneuvers	
Participants	1st attempt	2nd attempt	1st attempt	2nd attempt
1	1	2	2	2
2	1	2	2	2
3	2	2	2	2
4	2	2	2	2
5	2	2	1	2
6	2	2	2	2
7	2	2	2	1
8	1	2	1	2
9	2	1	2	2
10	1	2	1	2
11	2	2	2	2
12	2	2	2	2
13	2	2	1	2
14	2	2	2	2
15	2	2	2	2

Results of the Epley and Semont maneuvers performed by the group of ENT practitioners before and after using the labyrinth model, expressed as the number of steel balls (from 0 to 2) brought to the labyrinth.

Table S5. Medical students' answers to the satisfaction survey

Participants	Question 3	Question 4	Question 5	Question 6	Question 7
1	2	5	5	4	All
2	1	5	5	4	All
3	1	5	5	5	All
4	1	4	5	5	Med
5	3	4	5	4	All
6	2	5	5	4	All
7	3	5	5	4	All
8	2	5	5	4	All
9	2	4	5	5	ENT
10	2	5	5	4	All
11	2	5	4	5	All
12	1	5	2	4	EM, ENT
13	1	4	4	3	All
14	3	4	5	4	All
15	1	5	5	3	All

Table S6. EM residents' answers to the satisfaction survey

Participants	Question 3	Question 4	Question 5	Question 6	Question 7
1	3	5	5	4	All
2	5	5	5	4	All

3	3	5	5	5	All
4	2	5	5	4	EM
5	4	5	5	3	All
6	5	5	5	5	All
7	4	5	5	5	Med, ENT
8	3	5	5	4	All
9	3	5	5	4	All
10	3	5	4	4	All
11	3	5	4	4	Med, ENT
12	3	5	4	5	All
13	3	5	5	5	All
14	2	4	4	4	All
15	3	4	4	4	EM

Table S7. ENT residents' answers to the satisfaction survey

Participants	Question 3	Question 4	Question 5	Question 6	Question 7
1	3	4	5	5	All
2	5	5	5	5	All
3	2	4	5	4	All
4	2	5	5	5	All
5	2	5	5	4	All
6	4	4	5	5	Med, EM
7	4	4	4	3	EM, ENT
8	5	5	4	5	ENT
9	4	5	5	4	All
10	3	5	4	5	All
11	1	5	4	4	Med, ENT
12	3	5	4	3	All
13	1	5	4	4	All
14	3	5	5	4	All
15	5	5	5	5	Med, EM

Table S8. ENT practitioners' answers to the satisfaction survey

Participants	Question 3	Question 4	Question 5	Question 6	Question 7
1	4	5	5	5	All
2	3	5	4	4	All
3	5	5	5	5	All
4	3	5	4	4	All
5	4	5	5	5	All
6	3	4	5	5	All
7	5	3	5	5	All
8	4	3	5	5	All
9	5	5	5	5	All
10	5	5	5	5	All
11	5	3	5	5	All
12	3	4	4	5	All
13	4	4	5	5	All
14	5	4	5	5	All
15	5	4	5	5	All
